# Section 12 of the National Insurance Act : A Year's Experience

**Published:** 1915-01-30

**Authors:** J. B. Cornish

**Affiliations:** Hon. Secretary. West Cornwall Dispensary and Infirmary, Penzance.


### Section 12 of the National Insurance Act: A Year's Experience.

*To the Editor of* The Hospital.

Sir,—From the beginning we have done our best to get the benefit of the provisions of Section 12 of the National Insurance Act, and now that we have had a full year's experience the results may be of interest to your readers.

Many reasons have hindered us from getting the full amount of the sickness benefit; in some cases there has been difficulty in getting the Society to agree in time; in some the patients have been really in need of the money; and in several our standing agreement with the Society is that we take two-thirds and allow the patient one-third on leaving. After all drawbacks we find that the number of the patients insured and without dependants has been 22 per cent. of the total admissions for the year.

The number for whom we have received the whole or part of the sickness benefit has been 11.8 per cent. We have received on the average £1 13s. 4d. for each of these. The total receipt represents £2⅔ per cent. of our total income for the year.

This amount is all to the good, and is far more than anything we have lost in consequence of the Act. I may add that almost without exception I have found the patients quite willing that the hospital should have the money.—Yours truly, J. B. Cornish, Hon. Secretary.

West Cornwall Dispensary and Infirmary, Penzance.
January 20, 1915.

[The experience of this infirmary, with its twenty-six beds, is certainly interesting, and we shall be glad to publish similar records of how institutions have fared under this section.—Ed. The Hospital.]

